# Insights into the Canine Blood Donor Experience: A Multicenter Study on Physiological and Behavioral Changes

**DOI:** 10.3390/vetsci12090876

**Published:** 2025-09-10

**Authors:** Rebecca Dini, Chiara Agnoli, Chiara Mariti, Eleonora Gori, Kateryna Vasylyeva, Michele Tumbarello, Veronica Marchetti

**Affiliations:** 1 Department of Veterinary Sciences, Veterinary Teaching Hospital “Mario Modenato”, University of Pisa, Via Livornese Lato Monte, San Piero a Grado, 56122 Pisa, Italyeleonora.gori@unipi.it (E.G.); veronica.marchetti@unipi.it (V.M.); 2 Department of Veterinary Medical Sciences, University of Bologna, Ozzano dell’Emilia, 40062 Bologna, Italy; chiara.agnoli2@unibo.it (C.A.); kateryna.vasylyeva2@unibo.it (K.V.); michele.tumbarello3@unibo.it (M.T.)

**Keywords:** dog, cortisol, stress, owner, blood donation, behavior

## Abstract

Blood donor welfare is extensively studied in humans, while limited data exist for blood donor dogs. This multicentric prospective study aimed to evaluate physiological and behavioral responses in 89 canine blood donors. Clinical parameters (heart rate, respiratory rate, blood pressure, rectal temperature) and serum cortisol levels were measured before and after blood donation (BD), along with owner-reported behavioral assessments. Results showed no significant changes in cortisol concentrations pre-BD/post-BD or in most of the clinical parameters, except for rectal temperature that was significantly increased after BD [38.5 °C (38.2–39.4 °C) vs. 38.6 °C (37.6–41.7 °C), *p* < 0.001] suggesting a transient response likely due to physical restraint. No significant differences were found between first-time and repeat donors. Owners reported higher signs of arousal during the pre-donation phase, indicating anticipation as key to the stress response. However, more than 80% of the owners reported that their dogs behaved normally after the procedure and throughout the BD day. These findings suggest that canine whole BD is generally well tolerated and unlikely associated with systemic physiological stress. However, increased attention to the anticipatory phase, along with careful temperature monitoring, individualized restraint techniques, and optimized environmental management, may further improve canine blood donor welfare.

## Simple Summary

While blood donor welfare is well documented in human medicine, limited data are available regarding canine blood donors. This multicentric prospective study investigated potential stress in dogs undergoing whole blood donation through clinical parameters and serum cortisol levels before and after the procedure. In addition, owner-reported behavioral assessments were collected. Findings showed no significant changes in serum cortisol pre-/post-blood donation, as well as for the majority of clinical parameters. An increase in rectal temperature following donation was registered. Owners reported behavioral signs of arousal more frequently during the pre-donation phase compared to during or after the procedure. Overall, the results suggest that canine blood donation is generally well tolerated. However, enhanced attention to the preliminary phase, individualized handling approaches and temperature monitoring may further support donor welfare and minimize stress-related responses.

## 1. Introduction

Blood donation plays a central role in both human and veterinary medicine, ensuring a stable supply of blood products needed to support a wide range of medical conditions for recipients requiring a transfusion. In human healthcare systems, blood donation is predominantly based on volunteering and although blood donation is generally perceived as a fulfilling and rewarding experience, physiological and psychological acute stress responses associated with blood donation are reported [1]. These stress-related changes occurring in the course of blood donation have been largely studied through the assessment of both clinical parameters and laboratory values [2,3,4,5,6]. For this reason, increasing attention has been placed on the welfare of human blood donors, not only to ensure their safety during the donation process but also to promote blood donor retention and long-term engagement in blood donor programs. Various strategies have been developed to make the blood donation experience as minimally stressful as possible, including environmental modifications and donor education [7,8]. These interventions aim to reduce the psychological impact of blood donation, optimize donor management, and ultimately strengthen donor compliance and improve donor retention.

Studies on the impact of blood donation-related stress in veterinary medicine are scarce. Canine and feline blood donation is generally considered a well-tolerated procedure. Acute and delayed stress responses, such as general weakness, tachycardia/tachypnoea, and localized cutaneous reactions (e.g., hematomas or skin irritation at the venipuncture site), have been observed only occasionally and thought to be less common than those observed in human donors [9,10]. Well-established protocols, such as the presence of the owner, environmental enrichment, involvement of trained veterinary staff, and, when necessary, the use of mild sedation, are suggested to mitigate both physiological and behavioral stress responses in animal blood donors [9]. There is much veterinary literature on canine stress-related response. This includes the measurement of serum, salivary and hair cortisol, used as a biomarker of the hypothalamic–pituitary–adrenal axis response. In combination with behavioral changes, cortisol concentration provides valuable insight into stress levels in dogs [11,12]. However, the lack of comprehensive studies on behavioral and physiological markers of stress, such as cortisol evaluation, in canine blood donors, along with the owner perception, highlights the need for further investigation to ensure donor welfare and optimize veterinary transfusion medicine.

This multicentric study aimed to assess physiological changes in dogs before and after blood donation through the measurement of both clinical parameters and serum cortisol levels. In addition, owner-reported observations of stress-related changes were registered to provide qualitative insights into the donor’s behavioral response.

## 2. Materials and Methods

This is a prospective multicentric study on client-owned canine blood donors conducted between June 2024 and March 2025, in the Teaching Veterinary Hospitals of University of Pisa and University of Bologna (Italy). The present study was received favorably by the Animal welfare Board of the University of Pisa (approval number 22/2024).

### 2.1. Blood Donor Dogs: Enrollment and Sub-Grouping

The dogs included in this study were part of blood donor programs of both Pisa and Bologna veterinary facilities. They were selected according to the Italian blood donor guidelines available at the time of this study. As required by the guidelines, dogs needed to be clinically healthy, aged between 2 and 8 years, and without abnormalities on hematological and biochemical bloodwork. They also needed to be negative for the most common endemic hemopathogens [13]. Age, sex, breed, and total number of blood donations (BDs) previously performed were retrieved from clinical records. Based on the total number of performed BDs, blood donor dogs were divided into three groups: novice (1–2 total BDs), intermediate (3–5 total BDs), and experienced (>5 total BDs).

### 2.2. Pre- and Post-Donation Clinical Parameter Recording and Sample Processing

On the day of the scheduled blood donation, each of the blood donor dogs underwent a clinical examination where rectal temperature (T), heart rate (HR), respiratory rate (RR) and non-invasive systolic (SYS), mean (MAP), and diastolic (DIA) blood pressure were measured and recorded at the pre-donation timepoint (T0). Rectal temperature was measured with a digital thermometer. Blood pressure was measured using the Pettrust device (BioCare Corp., Taoyuan City, Taiwan) for dogs enrolled at the University of Pisa, and the Suntech Vet25 (SunTech Medical Inc., Morrisville, NC, USA) for those enrolled at the University of Bologna.

Venipuncture of cephalic/saphenous vein was then performed by trained medical personnel to collect blood into EDTA and serum tubes for routine pre-donation blood work. Once deemed eligible for donation, the dog and owner were welcomed in an air-conditioned room dedicated for blood donation, where the dog was subsequently placed in either left or right lateral recumbency. A patch of hair was shaved over the left or right jugular region, depending on the recumbency position. The area was then disinfected, and blood was collected by gravity into a collection bag. The final 3–5 mL of the collected blood, remaining in the patient blood bag connection tube, was transferred into a serum tube. Right after the blood donation (T1 timepoint), the overall duration of the procedure (from needle insertion into the jugular vein to its removal), T, HR, RR, and SYS, MAP, DIA were registered.

Pre- and post-donation serum from donor dogs enrolled at the University of Pisa was analyzed at the internal Clinical Pathology Laboratory to assess serum cortisol (AIA-Pack Cortisol, Tosoh, Tokyo, Japan) using an automated enzyme immunoassay analyzer (AIA-900, Tosoh, Tokyo, Japan) [14]. Surplus pre- and post-donation fresh serum of donors from the University of Bologna was aliquoted in Eppendorf tubes and stored at −80 °C for up to 5 months and then transported under temperature-controlled conditions to the Clinical Pathology Laboratory of the University of Pisa, where they were analyzed using the same equipment and methods.

### 2.3. Owner-Reported Behavioral Assessment via Questionnaire

Owners of canine blood donors were invited to complete an online questionnaire, administered via Google Forms, in order to report their perceptions about behavioral changes exhibited by their dogs in relation to the blood donation process (Italian and English versions of the questionnaire are attached in Appendix A). The questionnaire was structured into three sections (a, b, c), each addressing a different aspect of the dog’s behavioral response. Section (a) aimed to evaluate the level of dog stress perceived by the owner in relation to three specific timepoints of the donation experience: before the procedure, during the procedure, and immediately after. Owners were asked to use a Likert scale assigning a score from 1 to 5 (1 = not at all; 2 = slightly; 3 = moderately; 4 = very; 5 = extremely) to express the level of arousal of their dog at each of these stages. Section (b) focused on identifying any behavioral change observed during the whole donation process and asked owners to indicate whether their dog showed signs of agitation/restlessness, fear, sociability/cheerfulness, or no changes at all. Section (c) explored whether the dog appeared to exhibit unusual behaviors such as reduced alertness, increased fatigue or tendency to sleep more, increased appetite, increased excitability, general restlessness possibly accompanied by vocalization, or whether the behavior throughout the day of the blood donation remained the same as other days. All responses were based on the owner’s subjective perception and direct observation of their dog’s behavior during the specified periods.

### 2.4. Statistical Analysis

Statistical analyses were performed using three commercial statistical software (SPSS version 23, IBM Corp, Armonk, NY, USA; Prism 7, GraphPad Prism, Boston, MA, USA; Rstudio version 12.0, Posit, Boston, MA, USA). Continuous data, such as age, clinical parameters (T, HR, RR, SYS, MED, and DIA) and serum cortisol, are reported as median and range if non-normally distributed, and as mean ± standard deviation (SD) if normally distributed. Normality distribution was assessed using the Kolmogorov–Smirnov test. Categorical variables were reported as percentage (%).

Each clinical parameter and serum cortisol concentrations were compared between timepoints T0 and T1 using Wilcoxon matched-pair signed rank test or paired t-test, depending on the distribution. Afterwards, based on the experience of blood donor dogs, clinical parameters and serum cortisol levels were compared among the novice/intermediate/experienced blood donor groups using Kruskal–Wallis test. For this specifical analysis, a delta (∆) was calculated between the parameter at T1 minus the same parameter at T0 (e.g., ∆cortisol = T1 cortisol − T0 cortisol). The potential influence of season of BDs (summer-/winter-like temperatures) on clinical parameters and serum cortisol was assessed by comparing data collected in the two broad periods using Mann–Whitney U-test.

Friedman rank sum test and subsequent pairwise comparisons, using Conover’s all-pairs test for a two-way balanced complete block design with Bonferroni correction, were used to compare owner-reported behavior for dogs at different timepoints (before, during and after BD). All *p*-values were considered statistically significant when *p* < 0.05.

## 3. Results

### 3.1. Blood Donor Dogs: Clinical Parameters and Cortisol Concentrations Pre-/Post-Donation

Seventy-nine blood donor dogs were enrolled. The median age was 6 years (range 2–8 years), and 30 (37.9%) dogs were female (14 were spayed, 16 intact) and 49 (62.1%) were male (21 neutered, 28 intact). The majority of dogs were mixed breed (*n* = 27, 34.2%), followed by Labrador Retrievers (*n* = 12; 15.2%), Golden Retrievers (*n* = 7; 8.9%), Weimaraners (*n* = 6; 7.6%), German Shepherds (*n* = 5; 6.3%), Maremma Shepherds (*n* = 5, 6.3%), Bernese Mountain dogs (*n* = 3, 3.8%), Great Danes (*n* = 3, 3.8%), Rhodesian Ridgebacks (*n* = 2, 2.5%), Cane Corso dogs (*n* = 2; 2.5%), and Boxer dogs (*n* = 2; 2.5%). The was one each of the following breeds: American Staffordshire Terrier, Dogo Argentino, Flat-Coated Retriever, Dobermann, and Greyhound. The median number of total BDs performed by the enrolled dogs was 5 (range 1–15). The duration of the BD procedure was registered in 68 dogs, and the median duration was 9 min with a range of 3–20 min.

There were no statistically significant differences in HR, RR, SYS, MAP, DIA and serum cortisol levels between timepoints T0 and T1, while rectal temperature at T1 was significantly higher than at T0 (*p* < 0.001). A comparison of the clinical parameters and serum cortisol concentrations at the two timepoints is reported and summarized in Table 1. Data are presented as median (range) or mean ± standard deviation (SD), depending on the normality of distribution, along with corresponding *p*-values.

A total of 18 dogs (22.8%) were categorized as novice blood donors, 23 (29.1%) dogs were classified as intermediate blood donors, and 38 (48.1%) were considered experienced blood donors. None of the clinical parameters (T, HR, RR, SYS, MAP, and DIA) nor serum cortisol levels significantly differed among the novice/intermediate/experienced blood donor groups (each *p* > 0.05; Table 2).

A total of 27 (34.2%) blood donations were performed when temperatures are likely to be high (June–September), while 52 (65.8%) were conducted during winter-like temperatures (October–March). Season-like temperatures did not significantly influence serum cortisol levels or clinical parameters, except for RR, which was significantly higher during summer-like temperatures than during colder temperatures. Specifically, median RR at T0 timepoint was 60 bpm (range 28–90) in summer-like temperatures, whereas this was 38 bpm (range 12–90) in winter-like temperatures. (*p* = 0.005). Median T1 RR was 60 bpm (range 24–90) in summer-like temperatures, while median T1 RR was 38 bpm (range 17–90) in winter-like temperatures (*p* = 0.014).

### 3.2. Owner-Reported Behavioral Changes in Blood Donor Dogs

**Figure 1:**
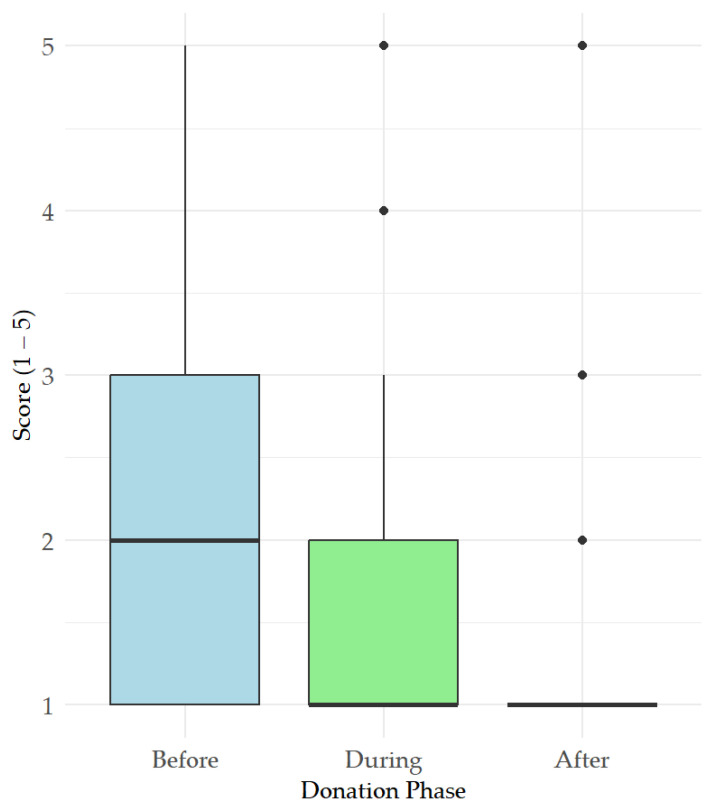
Owner-reported behavioral signs of arousal using a Likert Scale (score system 1–5; 1 = not at all; 2 = slightly; 3 = moderately; 4 = very; 5= extremely) before/during/after BD.

The results of dog behavioral signs of arousal, as assessed by owners using a Likert scale, in relation to specific timepoints (before, during, and after donation) are summarized in Figure 1, showing a clear trend of reduction throughout the BD procedure. In more detail, owners scored their dogs as more aroused before the BD than during (t = 4.35, *p* < 0.001) and after the procedure (t = 7.72, *p* < 0.001), as well as more aroused during the BD than after it (t = 3.36, *p*= 0.01).

According to the owners’ responses, 41/79 (51.9%) blood donor dogs were perceived as more sociable/cheerful, 20/79 (25.3%) dogs as fearful, and 18/79 (22.8%) dogs as agitated/restless during the whole blood donation process. None of the blood donor dogs were perceived by their owner as behaving normally.

Based on owner-reported questionnaire responses, 67/79 blood donor dogs (84.8%) were perceived as behaving normally throughout the day they donated. However, 7/79 (8.9%) dogs were reported to show increased fatigue/greater tendency to sleep, 4/79 (5.1%) dogs were perceived as hungrier than usual, and 1 dog (1.3%) was described as generally restless.

## 4. Discussion

Donor welfare has become a central focus of research in human transfusion medicine, where blood donation is majorly based on voluntary and altruistic participation. Considering this context, ensuring the safety and wellbeing of blood donors is essential, not only for ethical reasons, but also to promote donor retention and trust in donation programs. In human medicine, several studies have investigated how blood donation may induce physiological and stress-related changes, while in veterinary medicine the scientific literature is still scarce. In human medicine, most studies focused on the influence of blood donation on cardiovascular and endocrine parameters. The evaluation of HR and arterial blood pressure—SYS, MAP, and DIA—before and after donation has yielded conflicting findings [15]. While HR often remains stable throughout the procedure [15], arterial pressures have been reported to either increase [15,16,17,18], remain unchanged [4,19], or, in some cases, decrease pre-donation [5] compared to post-donation. The results of Kaur et al., who reported significantly higher arterial pressure and state anxiety scores in donors prior to venipuncture than following the donation, might suggest that the pre-donation period may represent the most critical phase in terms of physiological and psychological stress [15]. In the current study, no significant changes were observed comparing HR, blood pressure, and serum cortisol concentrations before and after blood donation in dogs. It is noteworthy that, although no significant difference was observed between median SYS values before and after blood donation, both measurements exceeded the reference range of healthy dogs (SYS < 140 mmHg) established by ACVIM guidelines [20]. According to the authors, systolic blood pressure may represent a parameter worth monitoring more closely, including through repeated measurements after donation and potentially even in the home environment, to help determine whether elevated values could possibly be more influenced by the veterinary/unfamiliar setting rather than the donation procedure itself.

Considering the behavioral signs of arousal, as assessed by dog owners, the high levels of arousal reported pre-donation and the statistical reduction observed throughout the BD procedure suggests that donation is not stressful for dogs. This finding aligns with the results of a questionnaire administered to dog owners to assess impaired welfare in their dogs during the different phases of a routine clinical visit, which showed that over 70% of dogs exhibited owner-perceived signs of stress during the waiting period before the actual clinical visit [21]. This might suggest that the anticipatory phase would need greater attention in the veterinary setting, with a focus on enhanced donor monitoring and improvements in environmental and procedural management strategies. The limited impact of BD on dogs’ welfare is strengthened by the behavior reported by owners for their dogs during the whole BD day. Although the percentage of dogs showing signs of long-term impairment is low, attention has to be paid to protect their wellbeing and, more generally, that of individual dogs who might experience BD differently, as suggested by the variety of behavioral changes observed by owners during the procedure.

Among the evaluated parameters, rectal temperature was the only variable that significantly increased immediately after blood donation, regardless of room and outside temperatures. Similar findings were reported in human donors by Mora-Rodriguez et al. (2012), who observed a modest increase in rectal temperature two hours post-donation at rest [22]. These changes are thought to reflect mild thermoregulatory alterations possibly mediated by sympathetic nervous system activation or transient release of inflammatory mediators during donation [22]. Notably, in the veterinary setting, physical restraint is required to safely perform venipuncture and ensure immobilization during donation. This may have a transient impact on the dog: if poorly tolerated by the animal, physical restraint may induce transient arousal and possibly a negative emotional response. This can lead to an increase in respiratory rate and core body temperature. This reaction could result in a tighter restraint, potentially creating a vicious circle. Remarkably, the observed increase in rectal temperature appeared to be independent of room conditions, as the donation room was air-conditioned and maintained at a constant temperature. However, environmental thermal influence more broadly might have a negative effect, as higher temperatures were recorded during periods with higher seasonal temperatures.

The lack of a significant change in serum cortisol concentrations before and after donation in our study supports this interpretation, suggesting that the procedure itself was not associated with a systemic stress response, and that the observed physiological changes may instead be related to the animal’s response to physical restraint. In contrast, in human studies, it is suggested that blood donation is associated with activation of the hypothalamic–pituitary–adrenal axis. Hoogerwerf et al. demonstrated significantly elevated salivary cortisol levels immediately prior to venipuncture compared to post-donation values, indicating that anticipatory stress plays a major role in the cortisol response [23]. This study aligns with the results of a previous work by Bellitti et al., who found higher plasma cortisol concentrations in first-time donors prior to blood collection, which decreased significantly after donation, while experienced donors exhibited a blunted or absent cortisol response [4]. In contrast to these human medicine findings, our study in canine blood donors did not reveal any significant differences in serum cortisol levels or in most clinical parameters between pre- and post-donation timepoints. Furthermore, no significant differences were observed in either clinical parameters or cortisol levels between first-time and experienced donors. These results may suggest that whole blood donation is generally well tolerated by dogs, regardless of their previous donation experience.

Considering rectal temperature was the only parameter that was significantly altered immediately after blood donation, regardless of season/room temperature, the authors recommend implementing improved restraint strategies and environmental enrichment to better manage potential physiological responses in donor dogs. Furthermore, considering that one outlier presented with marked hyperthermia (41.7 °C) immediately post-donation, routine measurement of rectal temperature is recommended as part of standard monitoring protocols in canine blood donors.

This study presents several limitations to be acknowledged when interpreting the findings. First, the research was conducted across two independent transfusion centers. Although both institutions follow standardized protocols, variations in the working environment and differences in personnel involved in the blood collection process may have introduced variability in the animals’ stress responses. Although this limitation reflects the real-world context of clinical veterinary visits, not all dogs were handled by the same operators, potentially influencing behavioral and physiological outcomes due to differences in handling style, experience, and human–animal interaction. While most clinical parameters, such as heart rate, respiratory rate, and body temperature, were measured using the same methodology, blood pressure measurements may have been affected by procedural biases. Specifically, different devices were used across the two centers, and the cuff was not always placed on the same anatomical site (e.g., forelimb vs. tail), which may have introduced measurement bias. Finally, serum cortisol measurements may have been subject to minor fluctuations due to circadian rhythm; however, the veterinary literature remains inconclusive regarding the extent to which diurnal variation significantly affects cortisol levels in dogs [12,24,25,26]. Therefore, a minimal circadian influence cannot be entirely ruled out as a potential source of bias.

## 5. Conclusions

This prospective multicentric study suggests that canine whole blood donation is generally well tolerated, with no significant changes observed in heart rate, respiratory rate, blood pressure, or serum cortisol levels except for a mild but consistent increase in rectal temperature post-donation, likely related to handling and restraint rather than systemic stress. Owner-reported arousal of dogs was higher before donation compared to during or after the procedure, highlighting that anticipation is crucial for dog stress response. In addition, behavioral changes were reported to be transient and procedure-related. As future prospective research directions, improved restraint methods, environmental and behavioral management strategies, and routine temperature monitoring should be further investigated to enhance canine blood donor welfare, especially before blood donation.

## Figures and Tables

**Table 1:** Comparison between clinical parameters and cortisol levels measured before (T0) and immediately after donation (T1) with respective *p* value.

Parameter	T0	T1	*p* Value
T (°C)	38.5 (38.2–39.4)	38.6 (37.6–41.7)	**<0.001** ^a^
HR (ppm)	100 (76–150)	100 (60–150)	0.66 ^a^
RR (bpm)	45 (12–90)	48 (17–90)	0.88 ^a^
SYS (mmHg)	160 (±23)	152 (±22)	0.11 ^b^
MAP (mmHg)	113 (±22)	111 (±19)	0.22 ^b^
DIA (mmHg)	94 (54–146)	92 (41–138)	0.26 ^a^
Cortisol (mcg/dL)	2.2 (0.24–6.9)	2.6 (0.33–7.8)	0.12 ^a^

Legend: T: rectal temperature; HR: heart rate; RR: respiratory rate; SYS: systolic blood pressure; MAP: mean blood pressure; DIA: diastolic blood pressure. Statistical test used: ^a^ Wilcoxon test; ^b^ paired t-test. Statistically significant *p*-value is displayed in bold.

**Table 2:** Descriptive statistics of clinical parameters and serum cortisol level in blood donor dogs sub-grouped based on their experience (novice/intermediate/experienced).

Parameter		Novice	Intermediate	Experienced
T (°C)	T0	38.5 (37.6–39.0)	38.3 (32.2–39.4)	38.5 (37.9–39.3)
	T1	38.7 (37.7–41.7)	38.7 (37.7–39.4)	38.5 (37.6–39.5)
	∆	0.1 (−0.5–3.6)	0.2 (−0.6–5.5)	0.1 (−1.0–0.7)
HR (ppm)	T0	104 (80–150)	97 (80–148)	100 (76–150)
	T1	100 (70–130)	100 (60–160)	106 (75–150)
	∆	−8.0 (−32–40)	4.0 (−38–68)	3.5 (34–140)
RR (bpm)	T0	60 (17–90)	42 (12–90)	45 (20–90)
	T1	40 (17–90)	55 (18–90)	48 (20–90)
	∆	0.0 (−20–25)	0.0 (48–58)	0.0 (−46–70)
SYS (mmHg)	T0	158 (108–196)	165 (124–185)	160 (103–200)
	T1	150 (108–196)	165 (121–194)	150 (110–195)
	∆	−8.0 (−47–31)	−6.0 (−28–55)	−8.5 (−71–60)
MAP (mmHg)	T0	114 (74–153)	116 (84–150)	113 (67–153)
	T1	112 (74–150)	112 (75–150)	104 (67–140)
	∆	0.0 (−44–27)	−0.5 (−42–39)	−4.5 (−75–36)
DIA (mmHg)	T0	91 (54–132)	99 (71–142)	91 (60–146)
	T1	97 (55–135)	98 (50–138)	86 (41–120)
	∆	2.0 (−42–34)	−3.5 (−49–33)	−2.5 (−86–31)
Cortisol (mcg/dL)	T0	2.1 (0.9–6.1)	2.3 (1.0–5.7)	2.3 (0.2–6.9)
	T1	3.1 (0.7–6.1)	1.9 (0.3–7.3)	2.9 (0.5–7.8)
	∆	0.3 (−1.45–4.4)	−0.2 (−5.0–4.6)	0.2 (−3.7–5.3)

Legend: T: rectal temperature; HR: heart rate; RR: respiratory rate; SYS: systolic blood pressure; MAP: mean blood pressure; DIA: diastolic blood pressure; ∆: T1 parameter − T0 parameter. Statistical test used: Kruskal–Wallis test. All *p*-value were above 0.05.

## Data Availability

Data are available upon reasonable request due to other ongoing projects.

## Abbreviations

The following abbreviations are used in this manuscript:

BD	Blood Donation
T	Rectal Temperature
HR	Heart Rate
RR	Respiratory Rate
SYS	Systolic Blood Pressure
MAP	Mean Blood Pressure
DIA	Diastolic Blood Pressure
T0	Pre-donation Timepoint
T1	Post-donation Timepoint
SD	Standard Deviation

## Appendix A

The Italian version of the questionnaire for owners:

(1) Quanto è agitato il suo animale in relazione alle fasi di donazione? (Attribuisca un punteggio da 1 a 5: 1 = per niente/2 = poco/3 = abbastanza/4 = molto/5 = moltissimo)
  - Prima della donazione
  - Durante la donazione
  - Dopo la donazione
(2) Nota delle variazioni di comportamento del suo cane rispetto al comportamento abituale in relazione alle fasi di donazioni?
  - Agitazione/irrequietezza
  - Timore
  - Socievolezza/Allegria
  - Nessuna
(3) Nota comportamenti insoliti nella giornata in cui il suo cane viene sottoposto a donazione?
  - Abbattimento/ Fatica/tendenza a dormire di più
  - Aumento appetito
  - Vivacità/Ipereccitabilità/Vocalizzazioni
  - Comportamento abituale

The English version of the questionnaire for owners:

(1) How much is your animal aroused in relation to the donation phases? (Assign a score from 1 to 5: 1 = not at all/2 = slightly/3 = moderately/4 = very/5 = extremely)
  - Before the donation
  - During the donation
  - After the donation
(2) Do you notice any behavioral changes in your dog compared to their usual behavior during the whole donation procedure?
  - Agitation/restlessness
  - Fear
  - Sociability/Cheerfulness
  - No changes at all
(3) Do you notice any unusual behaviors on the day your dog undergoes donation?
  - Reduced alertness/Increase fatigue/tendency to sleep more
  - Increased appetite
  - Increased excitability/General restlessness/Vocalizations
  - Usual behavior
